# Response of Bacterial Metabolic Activity to the River Discharge in the Pearl River Estuary: Implication for CO_2_ Degassing Fluxes

**DOI:** 10.3389/fmicb.2019.01026

**Published:** 2019-05-29

**Authors:** Xiangfu Li, Jie Xu, Zhen Shi, Ruihuan Li

**Affiliations:** ^1^State Key Laboratory of Tropical Oceanography, South China Sea Institute of Oceanology, Chinese Academy of Sciences, Guangzhou, China; ^2^College of Marine Science, University of Chinese Academy of Sciences, Beijing, China

**Keywords:** bacterial production, bacterial respiration, bacterial growth efficiency, dissolved organic carbon, the Pearl River Estuary

## Abstract

Bacterial production (BP), respiration (BR) and growth efficiency (BGE) were simultaneously determined along an environmental gradient in the Pearl River Estuary (PRE) in the wet season (May 2015) and the dry season (January 2016), in order to examine bacterial responses to the riverine dissolved organic carbon (DOC) in the PRE. The Pearl River discharge delivered labile dissolved organic matters (DOM) with low DOC:DON ratio, resulting in a clear gradient in DOC concentrations and DOC:DON ratios. BP (3.93–144 μg C L^−1^ d^−1^) was more variable than BR (64.6–567 μg C L^−1^ d^−1^) in terms of the percentage, along an environmental gradient in the PRE. In response to riverine DOC input, BP and the cell-specific BP increased; in contrast, the cell-specific bacterial respiration declined, likely because labile riverine DOC mitigated energetic cost for cell maintenance. Consequently, an increase in bacterial respiration was less than expected. Our findings implied that the input of highly bioavailable riverine DOC altered the carbon portioning between anabolic and catabolic pathways, consequently decreasing the fraction of DOC that bacterioplankton utilized for bacterial respiration. This might be one of the underlying mechanisms for the low CO_2_ degassing in the PRE receiving large amounts of sewage DOC.

## Introduction

Estuaries are important sites for carbon cycling where the transport, transformation and removal of allochthonous and autochthonous organic matter occurs (Canuel and Hardison, 2016). Although estuaries only occupy 0.3% of global ocean area, approximately 4 × 10^14^ g C y^−1^ of organic matter is delivered to the ocean through estuaries (Hedges et al., 1997). CO_2_ degassing flux in estuaries is almost equal to CO_2_ uptake on the continental shelf that accounts for 7.2% of the global ocean area (Cai, 2011). It is speculated that CO_2_ fluxes to the atmosphere are higher in low-latitude estuarine waters due to high bacterial degradation of DOC induced by higher temperature and runoff loading, compared to high-latitude estuarine waters (Cai, 2011).

The Pearl River Estuary (PRE) is located in the tropical region and is eutrophic, and it receives on average 3.26 × 10^11^ m^3^ of freshwater each year (Zhao, 1990). Sewage delivers amounts of dissolved organic carbon (DOC) to the PRE via the runoff. As a result, DOC concentrations are extremely high (up to 5.68 × 10^3^ μg L^−1^) in the upper estuary (Harrison et al., 2008; He et al., 2010). Despite this, the CO_2_ degassing flux in the PRE is low (6.92 mol C m^2^ y^−1^), which is 5–26-fold lower than that for European estuaries (Borges et al., 2005; Zhai et al., 2007). Similarly, the same scenario also occurs in the eutrophic Hoogly Estuary in the northeast India. Physical processes are considered to be the reason for the surprisingly low CO_2_ flux in these heavily polluted estuaries (Shetye, 1999; Sundar and Shetye, 2005). To date, little is known on the potential mechanism for the low CO_2_ flux.

Heterotrophic bacteria play a key role in DOC transformation by converting DOC to bacterial biomass through the anabolic pathway and by decomposing DOC to CO_2_ and nutrients through the catabolic pathway (Fuhrman, 1992). The origin and composition of DOM regulate bacterial metabolic processes and community (Warkentin et al., 2011; Traving et al., 2017). Carlson et al. (2007) suggest that cell-specific bacterial respiration increases with the extent of environmental hostility since more energy is needed to safeguard metabolic flexibility in hostile environments. It is suggested that the quality of DOC plays a more important role in regulating carbon portioning between anabolic and catabolic processes than the supply of DOC in DOC-rich waters (Xu et al., 2013). An early study showed that sewage-derived DOC with a high fraction of carbohydrates and amino acids, which are among the most bioavailable fraction of DOC (Benner et al., 1992; Middelboe et al., 1995), accounted for 32–54% of estuarine DOC in the upper and middle part of the PRE (He et al., 2010). Hence, we hypothesized that labile DOC derived from sewage modulated carbon portioning between anabolic and catabolic processes, resulting in a greater fraction of carbon allocated for bacterial production (BP) and an increase in bacterial growth efficiency (BGE) in the PRE.

Previous studies were focused on variability in heterotrophic bacterial abundance or production and their relationship with environmental factors in the PRE (Liu et al., 2007; Zhou et al., 2011). However, bacterial respiration was never determined in the PRE due to its time-consuming nature. In our study, BP and respiration were measured simultaneously along the PRE in the wet (May 2015) and dry (January 2016) seasons, in order to examine the effect of riverine DOM input on carbon portioning between anabolic and catabolic processes, which would help us improve our understanding of the underlying mechanism for the surprisingly low CO_2_ outgassing in the PRE.

## Materials and Methods

### Study Sites and Sampling

**FIGURE 1:**
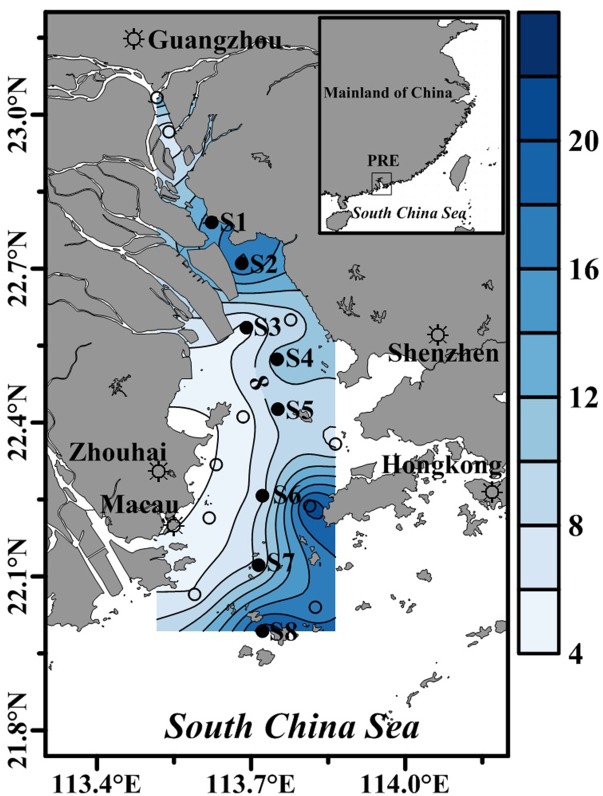
The location of sampling stations in the Pearl River Estuary during the two cruises. Bacterial metabolic rates, nutrients and Chl *a* concentrations were determined at stations denoted by solid circles, while water temperature and salinity were determined at all stations. The contour shows water depth.

Two cruises were conducted in May 2015 (wet season) and January 2016 (dry season). Water temperature and salinity were measured *in situ* with a YSI (556MPS, United States) at 18 stations (showed by circles in Figure 1) in the PRE, in eight (showed by solid circles in Figure 1) of which bacterial metabolism was determined, as well as nutrients and chlorophyll *a* (Chl *a*). The distance between S1 and S8 was about 92 km. The upper and lower reaches of the estuary are relatively deep (∼18 m) but shallow (∼8 m) in the middle reaches of the estuary (Figure 1). Water samples were collected from two depths at each station: 1 m below the surface and ∼3 m above the bottom.

Water samples for dissolved inorganic nutrients (NO_3_, NO_2_, NH_4_, PO_4_, and SiO_4_), DOC and total dissolved nitrogen (TDN) were filtered through pre-combusted (450°C for 4 h) glass fiber filters (GF/F). Nutrient samples were stored in acid-washed (10% HCl) plastic vials. DOC and TDN were stored in pre-combusted glass vials. Samples for Chl *a* were filtered onto the glass fiber filters (GF/F). All filtrates and filters were immediately frozen at −20°C until analysis in the laboratory.

Inorganic nutrient concentrations were determined manually with a spectrophotometer (Metash V-5000, China) following the protocols described by Grasshoff et al. (1999). NO_3_ was measured by the Zn-Cd column reduction method. NO_2_ and NH_4_ were analyzed by the diazonium compound coupled with a second aromatic amine and the indophenol blue color formations, respectively. PO_4_ was determined by the ascorbic acid method, and SiO_4_ was measured using molybdate, oxalic acid and a reducing reagent. DOC and TDN concentrations were simultaneously analyzed according to the high-temperature combustion method using a total organic carbon (TOC) auto-analyzer (Shimadzu TOC-LCPH, Japan) with TDN measuring unit (Alvarez-Salgado and Miller, 1998). The dissolved organic nitrogen (DON) concentration was calculated by subtracting the dissolved inorganic nitrogen (DIN) from the TDN. DIN was the sum of NO_3_, NO_2_ and NH_4_. The Chl *a* was extracted with acetone (90% v/v) in the dark for 24 h at 4°C before being analyzed using a fluorometer (Turner Designs Trilogy, United States) (Parsons, 1984).

The detection limits of nutrients were 0.05 μM (NO_3_), 0.02 μM (NO_2_), 0.03 μM (NH_4_), 0.02 μM (PO_4_), 0.10 μM (SiO_4_), and 4.00 μg C L^−1^ (DOC), which were significantly lower than our sample concentration. The ranges of precision estimates generally were 2–4%.

### Bacterial Abundance, Bacterial Production, and Bacterial Respiration

Water samples were successively filtered through a 20 μm nylon mesh and a 1 μm filter to remove larger particles and organisms. Samples for bacterial abundance (BA) were taken from the 20 and 1 μm filtrate, respectively. The samples (1 mL) for bacterial abundance were kept in cryovials, fixed with glutaraldehyde (final concentration 0.5%), and stored in liquid nitrogen until analyzed by a flow cytometer (Becton-Dickinson Accuri^TM^ C6, United States). Samples for bacterial numeration were performed following the method of Marie et al. (1997), were stained with 0.01% SYBR Green I in the dark for 15 min at room temperature before analysis and 1 μm beads were added. The data acquisition and analysis were directly performed with the Becton-Dickinson Accuri^TM^ C6 software, mainly detected using the plot of green fluorescence (FL1-A) versus side scatter (SSC) (Xu et al., 2013).

The 1 μm filtrate was filled into nine 60 mL BOD bottles for the measurement of bacterial respiration, three bottles of which were immediately fixed with Winkler reagents and determined for dissolved oxygen (DO). The other six bottles were incubated in the dark for 24 h and running water was used to maintain the surface *in-situ* temperature. At the end of the incubation, three of these bottles were used for the determination of DO, while the other three bottles were used for the determination of BA and BP. In addition, the samples for analysis of BP were also taken at the beginning of the incubation.

Dissolved oxygen concentration was titrated with an automated titration apparatus (Mettler-Toledo G20, Switzerland) which analyzed samples with a potentiometric detector to determine the titration endpoint (Oudot et al., 1988). A respiration quotient of 1.0 was used to convert BR into carbon units (Hopkinson, 1985; Sintes et al., 2010).

Bulk bacterial respiration (BR) was calculated by the following equation:

$$\text{BR}=\frac{({\text{DO}}_{\text{i}}-{\text{DO}}_{\text{f}})\times \text{RQ}\times 12}{\Delta \text{T}\times 32}$$

where *DO*_i_ and *DO*_f_ were the dissolved oxygen (μg L^−1^) determined at the beginning and the end of the incubation, and ΔT was the incubation time (d).

Bacterial production was measured with ^3^H-leucine incorporation according to the JGOFS protocol (Knap et al., 1996). ^3^H-leucine (final concentration 27 nM, specific activity 54.1 Ci/mmol) was added to 1 mL subsamples (quadruplicate) with one control fixed by cold trichloroacetic acid (TCA, final concentration of 5%). All subsamples were incubated in dark for 1 h, terminated by adding TCA and then stored at −20°C until analyzed. The incorporated ^3^H-leucine was determined using a microcentrifugation method as described in Kirchman (2001). After being centrifuged at high speed (15,000 r/min) and cleared by 5% TCA twice to remove supernatant, the microcentrifuge tubes were filled with 1 mL of scintillation cocktail and placed in the lab for 1∼2 days prior to measure using a liquid scintillation counter (Perkin-Elmer Tri-Carb 2810TR, United States). In the study area, where there was a clear environmental gradient due to input of the Pearl River with high nutrient and DOC levels, and a significant linear relationship between the leucine-to-carbon empirical conversion factors (CF) and salinity (*CF* = −0.02 *Salinity* + 1.54, *r*^2^ = 0.73, *p* < 0.01) was reported (Li et al., 2018). BP was estimated by multiplying the leucine incorporation rate by the CF that was calculated by salinity and the equation above in our study.

Bulk BP was calculated by the following equation:

$$\text{BP}=\frac{(BP_{\text{i}}+{\text{BP}}_{\text{f}})\times 24}{2}$$

where *BP*_i_ and *BP*_f_ are the BP (μg C L^−1^ h^−1^) determined at the beginning and the end of the incubation.

The cell-specific BP (sBP) and BR (sBR) were calculated using the following equation, respectively:

$$\begin{array}{c} \text{sBP}=\frac{2\times \text{BP}}{\left({\text{BA}}_{\text{i}}+{\text{BA}}_{\text{f}}\right)}\\ \text{sBP}=\frac{2\times \text{BR}}{\left({\text{BR}}_{\text{i}}+{\text{BA}}_{\text{f}}\right)} \end{array}$$

where *BA*_i_ and *BA*_f_ were bacterial abundance at the initial and the end of the incubation.

Bacterial carbon demand (BCD) was the sum of BP and BR, BGE was calculated according to the below equation:

$$\text{BGE}=\frac{\text{BP}}{\text{BP}+\text{BR}}=\frac{\text{BP}}{\text{BCD}}$$

### Statistical Analysis

A Pearson-test analysis was performed to determine significant differences between various variables (temperature, salinity, Chl *a*, DOC, the ratio of DOC:DON, bacterial abundance, BP, bacterial respiration, BCD and BGE) by SPSS (version 18) software. Type II linear regressions were performed to analyze the relationship between the variables using R software (version 3.5.2) with an “lmodel2” package. All variables were log-transformed before the regression analysis to fit the assumption of normal distribution and homoscedasticity.

## Results

### Temperature and Salinity

**FIGURE 2:**
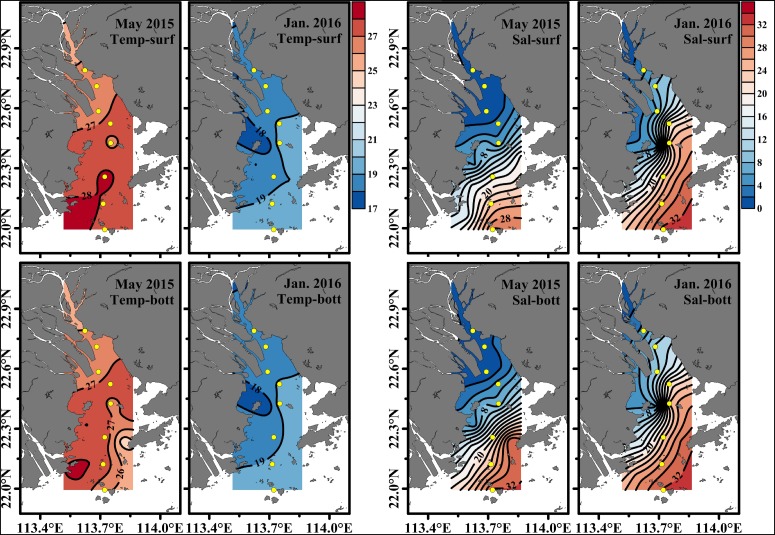
The horizontal distribution of temperature and salinity at the surface and the bottom in the Pearl River Estuary in the wet season (May 2015) and the dry season (January 2016). Temp, sal, surf, bott, and Jan represent temperature, salinity, surface, bottom, and January, respectively.

**FIGURE 3:**
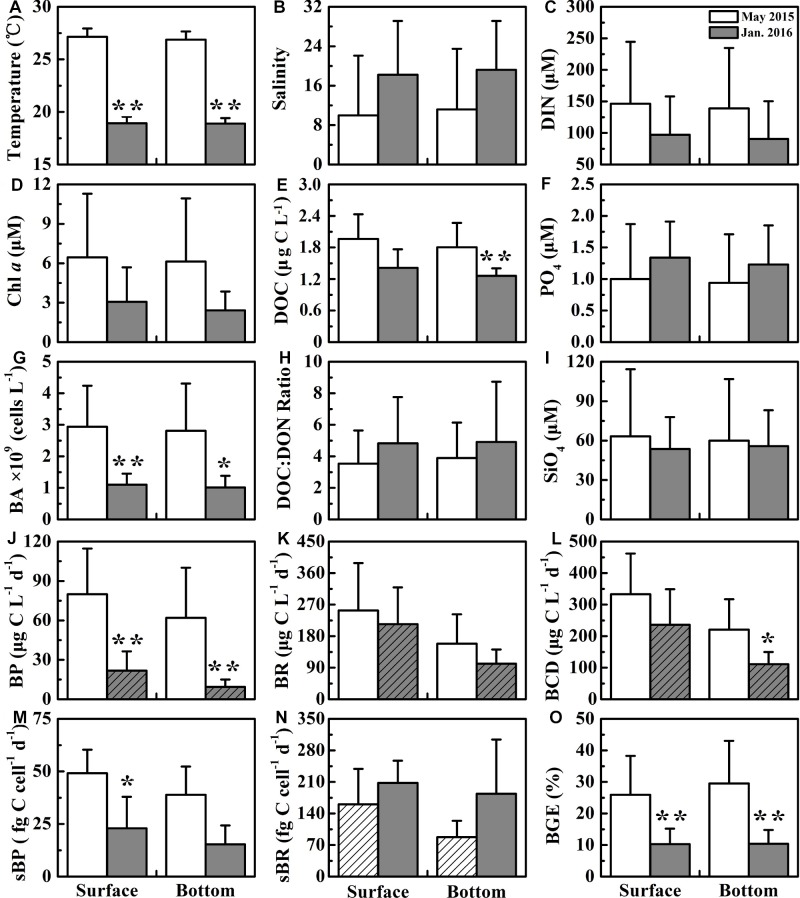
The average of variables at the surface and the bottom in the wet season (May 2015) and the dry season (January 2016). ^∗^ and ^∗∗^ denote that variables between the wet season and the dry season are on average significantly different at 0.05 (**p** < 0.05) and 0.01 (**p** < 0.01), respectively. The columns filled by oblique lines showing significant difference (**p** < 0.05) between the surface and the bottom in same season. The labels **(A–O)** are the number of each graph.

Surface seawater temperature and salinity generally increased from the upper to the lower estuary in May 2015 and January 2016. There was no significant difference in temperature and salinity between the surface and bottom layer in the upper estuary. However, a salinity stratification occurred in the middle and the lower estuary (from S4 to S8) during May 2015, and salinity in the surface layer was 1.65–2.38 lower than that in the bottom layer, while the water column was well mixed during January 2016. Moreover, an obvious salinity front occurred in the middle and upper estuary in May 2015 (near S5 and S6) and January 2016 (between S3 and S5) (Figure 2). Salinity was much lower in May 2015 (mean of 9.97 ± 12.1 at the surface and 11.2 ± 12.3 at the bottom) than that in January 2016 (average of 18.2 ± 10.9 at the surface and 19.2 ± 9.90 at the bottom) (Figure 3B). Temperature (27.1 ± 0.80°C at the surface and 26.9 ± 0.78°C at the bottom) in May 2015 was on average approximately 8°C higher than that (18.9 ± 0.62°C at the surface and 18.9 ± 0.52°C at the bottom) in January 2016 (*p* < 0.01, Figure 3A).

### Nutrients, DOC, and Chl *a*

**FIGURE 4:**
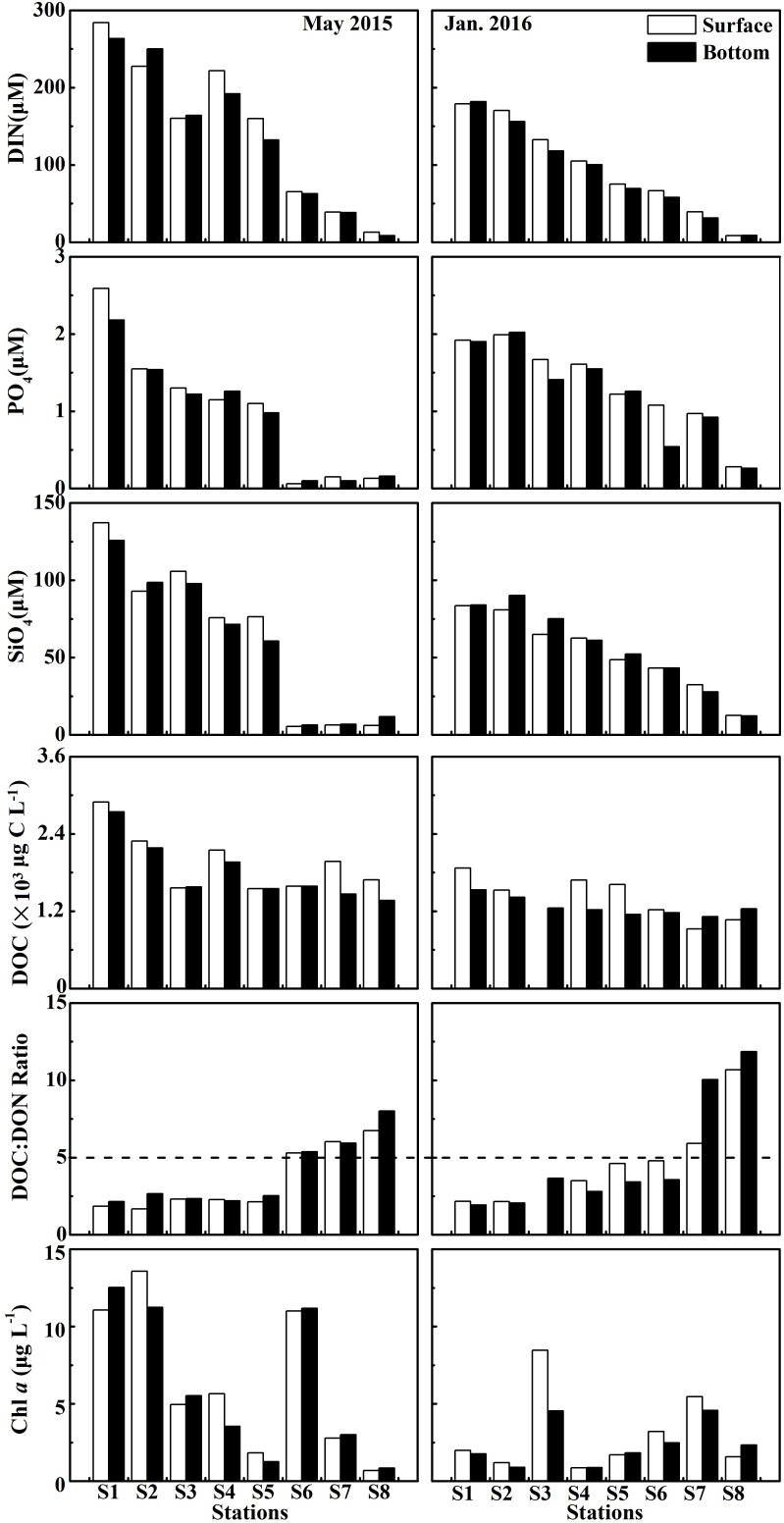
DIN, PO_4_, SiO_4_, DOC, Chl *a* concentrations and the ratio of DOC to DON at the surface and bottom at eight stations in the wet season (May 2015) and the dry season (January 2016). DIN = NO_3_ + NO_2_ + NH_4_.

DIN concentration decreased dramatically seawards from ∼300 μM at S1 to ∼11 μM at S8 in May 2015, from ∼180 μM at S1 to ∼9 μM at S8 in January 2016 (Figure 4). DIN concentration (mean of 146 ± 98.2 μM at the surface and 139 ± 95.8 μM at the bottom) in May 2015 was on average about 1.5-fold higher than that (mean of 97.1 ± 61.0 μM at the surface and 90.4 ± 60.0 μM at the bottom) in January 2016 (Figure 3C). PO_4_ was similar to the pattern of DIN, while PO_4_ concentration was disproportionately low (Figure 3F), with ∼1 μM in the upper and middle estuary and even down to ∼0.2 μM in the lower estuary (Figure 4). The variability in SiO_4_ was similar to the patterns of DIN, decreasing from 137 μM at S1 in May 2015 down to 5.36 μM at S8 in January 2016 (Figure 3I, 4).

DOC concentrations decreased seawards in these two seasons when the ratio of DOC:DON increased seawards (Figure 4). DOC concentration (the average of 1.96 ± 0.43 × 10^3^ μg L^−1^ at the surface and 1.80 ± 0.47 × 10^3^ μg L^−1^ at the bottom) in May 2015 was generally higher than that (1.42 ± 0.35 × 10^3^ μg L^−1^ at the surface and 1.26 ± 0.14 × 10^3^ μg L^−1^ at the bottom) in January 2016 (Figure 3E). However, the average ratio of DOC to DON in May 2015 (3.54 ± 2.10 at the surface and 3.90 ± 2.24 at the bottom) was slightly lower than that in January 2016 (4.83 ± 2.93 at the surface and 4.91 ± 3.82 at the bottom) (Figure 3H).

Chl *a* concentrations exhibited a seasonal variation, which were higher (the average of 6.45 ± 4.84 μg L^−1^ at the surface and 6.14 ± 4.80 μg L^−1^ at the bottom) in May 2015 than those (3.06 ± 2.63 μg L^−1^ at the surface and 2.41 ± 1.44 μg L^−1^ at the bottom) in January 2016 (Figure 3D). Chl *a* generally declined seawards along a salinity gradient in May 2015, except for the frontal zone where high Chl *a* concentrations (e.g., up to 11 μg L^−1^ at S6 in May 2015 and 4.54 μg L^−1^ at S3 in January 2016) occurred (Figure 4). The detailed datas of nutrients, DOC, and Chl *a* concentrations were shown in Supplementary Table 1.

### Bacterial Activity

**FIGURE 5:**
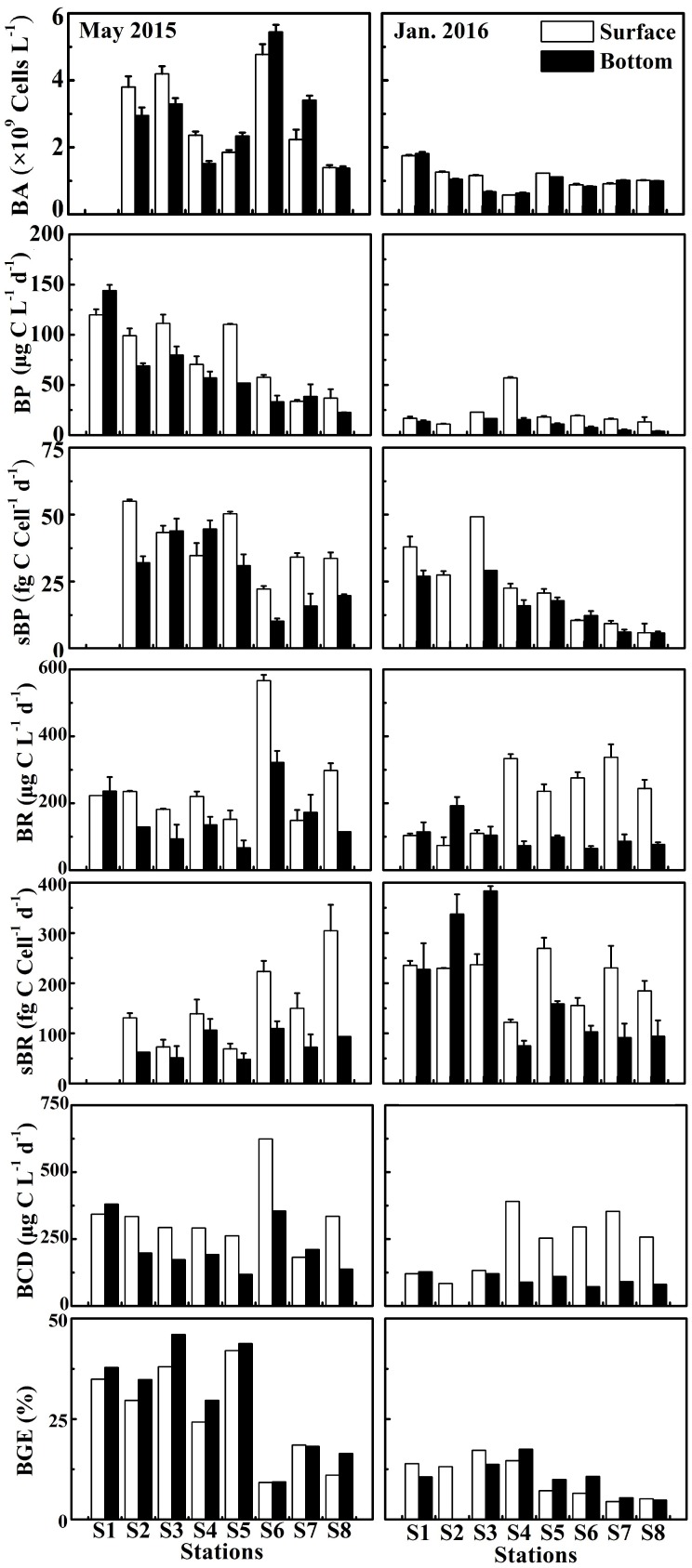
Bacterial abundance (BA), bacterial production (BP), cell-specific bacterial production (sBP), bacterial respiration (BR), cell-specific bacterial respiration (sBR), bacterial carbon demand (BCD), and bacterial growth efficiency (BGE) at the surface and bottom at eight stations in the wet season (May 2015) and the dry season (January 2016).

Bacterial abundance (BA) generally showed a declining trend along a salinity gradient (Figure 5). BA exhibited significant seasonal difference (*p* < 0.01 or *p* < 0.05), with a high in May 2015 (the mean of 2.94 ± 1.30 × 10^9^ cells L^−1^ at the surface and 2.81 ± 1.50 × 10^9^ cells L^−1^ at the bottom) and a low in January 2016 (1.10 ± 0.35 × 10^9^ cells L^−1^ at the surface and 1.01 ± 0.37 × 10^9^ cells L^−1^ at the bottom) (Figure 3G). However, there was no significant difference (*p* > 0.05) in BA between the surface and bottom in both seasons (Figure 3G). The highest BA occurred in the front (up to 2.88 ± 1.33 × 10^9^ cells L^−1^ at S6) in May 2015 (Figure 5).

Bacterial production (BP) in May 2015 and cell-specific bacterial production (sBP) in January 2016 declined from the upper to the lower estuary (Figure 5). In May 2015, BP (the average of 79.9 ± 34.7 μg C L^−1^ d^−1^ at the surface and 62.0 ± 38.1 μg C L^−1^ d^−1^ at the bottom) was significantly higher than that (*p* < 0.01, 21.8 ± 14.7 μg C L^−1^ d^−1^ at the surface and 9.42 ± 5.55 μg C L^−1^ d^−1^ at the bottom) in January 2016 (Figure 3J). sBP (49.1 ± 11.2 fg C cell^−1^ d^−1^) at the surface in May 2015 was significantly higher than that (*p* < 0.05, 23.0 ± 15.0 fg C cell^−1^ d^−1^) in January 2016 (Figure 3M).

The bulk bacterial respiration (BR) and the cell-specific bacterial respiration (sBR) exhibited different seasonal variations. BR (the average of 253 ± 135 μg C L^−1^ d^−1^ at the surface and 158 ± 83.6 μg C L^−1^ d^−1^ at the bottom) was slightly higher in May 2015 than that (214 ± 105 μg C L^−1^ d^−1^ at the surface and 101 ± 40.4 μg C L^−1^ d^−1^ at the bottom) in January 2016, while sBR was higher in January 2016 (208 ± 49.3 fg C cell^−1^ d^−1^ at the surface and 184 ± 120 fg C cell^−1^ d^−1^ at the bottom) than that (160 ± 78.5 fg C cell^−1^ d^−1^ at the surface and 87.6 ± 36.4 fg C cell^−1^ d^−1^ at the bottom) in May 2015 (Figure 3K,N). BR and sBR exhibited a dramatic spatial change of nine and eightfold along the estuary, respectively. Moreover, BR and sBR in the surface layer were relatively higher than those in the bottom at most stations (Figure 5).

Bacterial carbon demand (BCD) (the average of 333 ± 129 μg C L^−1^ d^−1^ at the surface and 221 ± 96.1 μg C L^−1^ d^−1^ at the bottom) was relatively high in May 2015 and low in January 2016 (236 ± 113 μg C L^−1^ d^−1^ at the surface and 111 ± 39.0 μg C L^−1^ d^−1^ at the bottom) (Figure 3L).

Bacterial growth efficiency (BGE) showed a seasonal pattern similar to BCD, which was significantly higher in May 2015 (*p* < 0.01, the mean of 26.0 ± 12.3% at the surface and 30.0 ± 13.5% at the bottom) than in January 2016 (10.3 ± 4.95% at the surface and 10.4 ± 4.42% at the bottom) (Figure 3O). BGE decreased from the upper to lower estuary in both seasons (Figure 5).

## Discussion

### Environmental Conditions

The hydrographic properties exhibited strong seasonal differences along the PRE owing to seasonal alterations in monsoon and river discharge. The PRE was dominated by freshwater under the prevalence of the southwest monsoon in May 2015, while the northeast monsoon prevailed in January 2016 and saline water from the South China Sea intruded into the PRE (Lu and Gan, 2015). As a result, temperature and salinity varied seasonally and spatially in the PRE.

Large amounts of anthropogenic sewage are put into the estuary with the Pearl River discharge, resulting in the enrichment of nutrients and DOC (Harrison et al., 2008). In our study, DOC concentration was generally high (9.24 × 10^2^ to 2.89 × 10^3^ μg L^−1^) in the PRE, in agreement with previous publications (1.01 × 10^3^ to 3.34 × 10^3^ μg L^−1^) in the same region (He et al., 2010). The average ratio of DOC to DON, as an indicator of the bioavailability of DOC (Kroer, 1993), was relatively low (4.28 ± 2.77) in the PRE, even lower than the value (∼6) in the sewage-rich Mandovi-Zuari estuary (Ram et al., 2007) and comparable to the elemental composition of bacterial biomass (∼5C:1N) (Goldman et al., 1987). Microbial preference for nitrogen-containing substrates has been reported (Kellogg and Deming, 2014). The relatively low ratio of DOC to DON provided evidence that most fractions of DOC were high bioavailability for bacterial activity in the PRE during our cruise.

**FIGURE 6:**
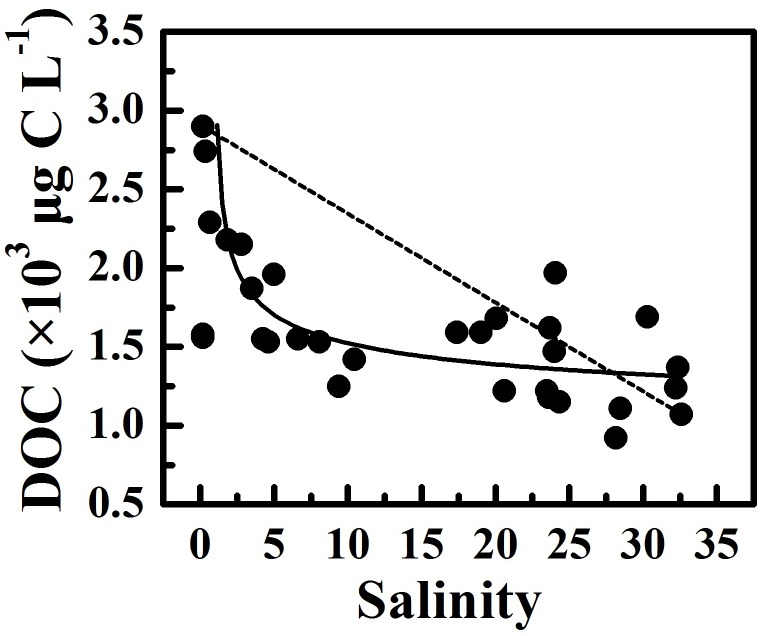
The variability of DOC concentration along salinity gradient during our cruises. The dashed line links the DOC concentration at the lowest and the highest salinity, and the solid line denotes the relationship between DOC concentration and salinity.

DOC concentrations decreased along the estuary, accompanied by an increase in DOC:DON ratios, suggesting that riverine DOC was labile and marine-derived DOC was refractory. Two end-member mixing models, where S1 with the lowest salinity was serviced as the freshwater endmember and S8 with the highest salinity was serviced as the seawater endmember, were used to assess the role of microbial degradation in reducing DOC. The DOC concentrations were derived from the conservative mixing line (Figure 6), suggesting that microbial degradation played a more important role than the dilution of seawater. The microbial degradation and the mixing of freshwater and saline water from the South China Sea caused an apparent gradient in DOC concentrations and DOC:DON ratios along the estuary. In addition, the resuspension of estuarine surface sediments releases relatively refractory organic matter to the surrounding water column (Komada et al., 2002). The sediment resuspension might replenish organic matter and cause an increase in DOC:DON ratios in January 2016 due to strong vertical mixing.

**FIGURE 7:**
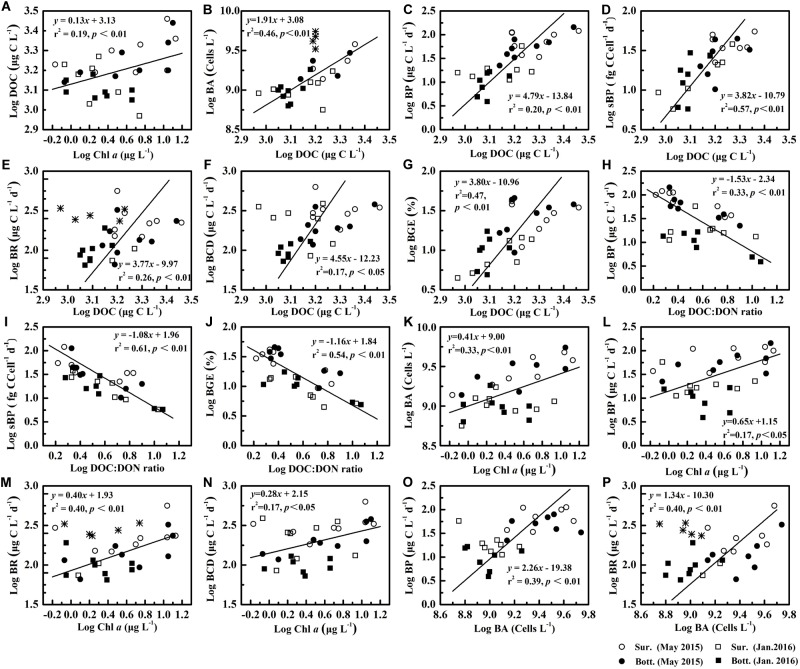
The relationship between various variables (Chl **a**, DOC, DOC:DON ratio, BA, BP, sBP, BR, BCD and BGE). 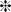 denotes the data being eliminated, including the BA in frontal area in May 2015 and BR at the surface in the lower reaches of the estuary during January 2016. The labels **(A–P)** are the number of each graph, and the equations and correlation coefficients between various variables are shown in Supplementary Table 3.

DOC concentration was positively significantly correlated with Chl *a* concentration (*r*^2^ = 0.19, *p* < 0.01, Figure 7A), implying that a part of bioavailable DOC resulted from phytoplankton in the PRE, which was consistent with findings that phytoplankton releases labile DOC (Shen et al., 2016). However, phytoplankton growth was often restricted by low light penetration due to high suspended particles in this estuary (Chen et al., 2004). Hence, riverine DOC should be a major source of DOC in the PRE, especially during the wet season or high flow periods (Ochs et al., 2010; Hitchcock and Mitrovic, 2015), which might explain why there was no correlation between Chl *a* concentration and DOC:DON ratio.

### Bacterial Metabolic Activity

Bacterial production varied over a wide range, rising even to an approximately 30-fold difference among sites and a fivefold difference between seasons, respectively. BP in our study was still in agreement with the ranges reported for other estuarine (from ∼10 to 180 μg C L^−1^ d^−1^) (Ochs et al., 2010) and coastal waters (from about 14.4 to 146 μg C L^−1^ d^−1^) (Lee et al., 2009). Spatiotemporal variability in BP was primarily attributed to riverine DOC input (*r*^2^ = 0.20, *p* < 0.01, Figure 7C), which favored the growth of metabolically active bacteria with high DNA in cells (Xu et al., 2013), resulting in an increase in BA and sBP. This suggestion was supported by a significant correlation between BA and DOC (*r*^2^ = 0.46, *p* < 0.01, Figure 7B), as well as between sBP and DOC (*r*^2^ = 0.57, *p* < 0.01, Figure 7D). Fluctuations in sBP might be the reflection of shifts in bacterial community composition, induced by alterations in the composition and quantity of DOM. It was speculated that spatial variations in BP were accompanied by shifts in bacterial community compositions. Coupling of BP and bacterial community composition was documented in other studies (Warkentin et al., 2011; Traving et al., 2017). Chl *a* concentration was significantly correlated with BA (*r*^2^ = 0.33, *p* < 0.01, Figure 7K) and BP (*r*^2^ = 0.17, *p* < 0.05, Figure 7L), respectively, since phytoplankton-derived DOC was labile (Shen et al., 2016), fueling bacterial growth. Bacterial activity is often reported to be coupled with phytoplankton biomass in other regions (Ochs et al., 2010). In contrast to the Chl *a* concentration, the stronger relationship of DOC concentration versus BA and BP, respectively, indicated that riverine DOC fueled bacterial growth, in addition to phytoplankton-derived DOC. Therefore, in response to riverine input, the enhancement of BP was the combined result of increased BA (*r*^2^ = 0.17, *p* < 0.05, Figure 7O) and sBP.

Bacterial respiration exhibited little spatiotemporal variability and was less variable than BP, likely due to carbon partitioning between catabolic and anabolic processes with changing environmental conditions. Our findings were in agreement with previous reports (Roland and Cole, 1999; del Giorgio and Cole, 2000). sBR reflects a physiological response of bacterial cells to environmental conditions on the cellular scale, which often incremented with increasing environmental hostility (Carlson et al., 2007). In May 2015, sBR increased seawards along the estuary (Figure 5), accompanied by an increase in DOC:DON ratio (Figure 4). Furthermore, sBR in May 2015 with high Chl *a* levels was lower than that in January 2016 (Figure 3N). Spatial and temporal variability in sBR was most likely induced by changes in the environmental hostility (i.e., quality and quantity of DOC) in the PRE. A decline in the quantity and quality of DOC changed carbon partitioning between anabolic and catabolic processes, which ultimately allocated more carbon to catabolic process for cell maintenance and repair rather than growth or division (Figure 7H,I) (del Giorgio and Cole, 2000). The input of the river discharge affected BR and sBR in contrasting ways, increasing the bulk BR by stimulating bacterial growth as reflected by a significant correlation of BR versus DOC and BA (Figure 7E,P) and reducing sBR likely due to mitigated energetic cost for cell maintenance in response to labile riverine DOC. Consequently, response of the bulk BR to changes in environmental conditions was always mitigated.

Bacterial carbon demand was less variable than BP along the estuary. Bacterial respiration contributed to on average 80.7 ± 12.9% of BCD, which was calculated by dividing BCD by BR for the discrete sample. The magnitudes of BCD in PRE in our study were generally close to that in its adjacent Daya Bay, China (Song et al., 2015). BCD was weakly but significantly correlated with DOC (*r*^2^ = 0.17, *p* < 0.05, Figure 7F) and Chl *a* concentrations (*r*^2^ = 0.17, *p* < 0.05, Figure 7N), respectively, suggesting that BCD increased with DOC supply, in agreement with previous findings of high BCD in substrate-rich or productive systems (Paerl et al., 2003; Apple et al., 2004). In the eutrophic PRE, DOC concentrations remained relatively high and varied in a relatively narrow range (1.36 × 10^3^ to 2.89 × 10^3^ μg L^−1^ in May 2015 and 9.24 × 10^2^ to 1.87 × 10^3^ μg L^−1^ in January 2016), which met 8 days bacterial growth, derived from daily BCD, indicating that bacterial metabolic activity was primarily regulated by the quality or bioavailability of DOC, rather than DOC concentration.

In our study, BGE ranged from 4 to 46%, with a high in the wet season (28 ± 13%) and a low in the dry season (10 ± 5%), which was in agreement with the range reported in the tropical Mandovi and Zuari estuaries (mean = 28 ± 14%) (Ram et al., 2007) and coastal waters of Peninsular Malaysta (ranged from 2 to 40%) (Lee et al., 2009). An early study suggests that BGE decreases with increasing temperature (Rivkin and Legendre, 2001). However, in our study, BGE in May 2015 with high temperature was significantly (*p* < 0.01, Figure 3O) higher than that in January 2016 with low temperature. Hence, variability in BGE in PRE was more likely linked to the quality and quantity of DOC, as indicated by a significant correlation between BGE and DOC concentrations (*r*^2^ = 0.47, *p* < 0.01, Figure 7G), as well as a stronger correlation between BGE and the ratio of DOC to DON (*r*^2^ = 0.54, *p* < 0.01, Figure 7J). In the dry season, when pristine seawater invaded the estuary, lower BGE was more likely caused by a decline in DOC concentrations and an increase in DOC:DON ratio. Similarly, Apple and del Giorgio (2007) find that the bioavailability of DOC plays a more important role in shaping bacterial carbon metabolism for a given DOC in a temperate salt-marsh estuary.

In the PRE, primary production remained low due to light limitation (Yin et al., 2004), which played a minor role in reducing CO_2_ emission through photosynthesis. Over a short residence time (less than 3 days) of the water column (Yin et al., 2000), bacterial growth might be primarily fueled by labile DOC since labile DOC was preferentially utilized by bacteria. DOC lability altered the carbon partitioning between catabolic and anabolic processes. Consequently, a small fraction of carbon utilized by bacteria was transformed to CO_2_, which might be partly responsible for low CO_2_ degassing in the DOC-rich PRE.

### Evaluation of the Experimental Approach

Bacteria in 1 μm-filtrate grew rapidly in the incubation bottles in dark, and bacterial abundance changed dramatically over 24 h (Supplementary Table 2), which was in parallel to the incubation for bacterial respiration samples. The bulk BP derived from the BP measured at the beginning and the end of the incubation minimized the underestimation of the daily BP and BGE. In this study, we focused on the response of production and respiration of bacterial community to changes in riverine DOC, rather than specific bacterial groups. Spatial variability in DOC along the estuary is accompanied by alteration in salinity. A recent study shows that bacterial metabolic activity is primarily regulated by substrate availability along the salinity gradient in coastal waters adjacent to PRE (Xu et al., 2018). The quantity and quality of DOC should play a key role in regulating bacterial metabolic activity in the PRE.

## Conclusion

The quality and quantity of DOC play a significant role in regulating bacterial metabolic activities. Two aspects (production and respiration) of bacterial metabolic activity responded differently to labile riverine DOC. BP increased dramatically, while an increase in bacterial respiration was much less than expected as the lability of riverine DOC likely lowered the energetic cost for cell maintenance, leading to a decrease in cell-specific bacterial respiration in response to the riverine DOC environment. Riverine DOC input altered carbon partitioning between the anabolic and catabolic pathways, reducing the fraction of DOC that bacterioplankton utilized for bacterial respiration, which was likely one of reasons for the low CO_2_ degassing fluxes in estuaries heavily influenced by sewage DOC.

## Author Contributions

XL performed the experiments and measured samples. RL and ZS helped in collecting and determining the some samples. XL and JX contributed to the data analysis and manuscript writing.

## Conflict of Interest Statement

The authors declare that the research was conducted in the absence of any commercial or financial relationships that could be construed as a potential conflict of interest.
