# Supplementary material for: Response of Bacterial Metabolic Activity to the River Discharge in the Pearl River Estuary: Implication for CO2 Degassing Fluxes
Source: Front Microbiol. 2019 May 29;10:1026. doi: 10.3389/fmicb.2019.01026 (PMC6548906; doi:10.3389/fmicb.2019.01026)
Supplement: Supplementary file 1 [file Table_1.DOCX]

Table 1 Temperature, salinity, DIN, PO_4_, SiO_4_, Chl *a* and DOC concentrations and the ratio of DOC to DON at the surface and bottom at eight stations in the wet season (May 2015) and the dry season (January 2016).

| Cruise | Stations | Sample layer | Water depth (m) | Temperature(℃) | Salinity | DIN (μM) | PO_4_ (μM) | SiO_4_ (μM) | DOC (×10^3^ μg C /L) | DOC:DON ratio | Chl *a*(μg/L) |
| --- | --- | --- | --- | --- | --- | --- | --- | --- | --- | --- | --- |
| May 2015 | S1 | Surface | 16.5 | 26.12 | 0.19 | 284.00 | 2.59 | 137.14 | 2.90 | 1.85 | 11.06 |
|  | S1 | Bottom | 16.5 | 26.01 | 0.36 | 263.39 | 2.18 | 125.71 | 2.74 | 2.15 | 12.53 |
|  | S2 | Surface | 18.5 | 26.82 | 0.66 | 227.55 | 1.55 | 92.86 | 2.29 | 1.67 | 13.58 |
|  | S2 | Bottom | 18.5 | 26.63 | 1.84 | 250.13 | 1.54 | 98.57 | 2.18 | 2.66 | 11.24 |
|  | S3 | Surface | 6.5 | 26.30 | 0.19 | 160.08 | 1.30 | 105.71 | 1.56 | 2.30 | 4.96 |
|  | S3 | Bottom | 6.5 | 26.30 | 0.18 | 163.95 | 1.22 | 97.86 | 1.58 | 2.34 | 5.53 |
|  | S4 | Surface | 9.0 | 27.51 | 2.78 | 221.79 | 1.15 | 75.71 | 2.15 | 2.28 | 5.66 |
|  | S4 | Bottom | 9.0 | 27.66 | 5.01 | 192.05 | 1.26 | 71.43 | 1.96 | 2.20 | 3.55 |
|  | S5 | Surface | 8.0 | 26.57 | 4.25 | 159.87 | 1.10 | 76.43 | 1.55 | 2.13 | 1.83 |
|  | S5 | Bottom | 8.0 | 26.63 | 6.63 | 132.16 | 0.98 | 60.71 | 1.55 | 2.52 | 1.26 |
|  | S6 | Surface | 7.5 | 28.29 | 17.38 | 65.30 | 0.06 | 5.36 | 1.59 | 5.30 | 11.01 |
|  | S6 | Bottom | 7.5 | 27.82 | 19.03 | 62.89 | 0.10 | 6.43 | 1.59 | 5.37 | 11.18 |
|  | S7 | Surface | 8.1 | 27.95 | 24.06 | 38.84 | 0.15 | 6.43 | 1.97 | 6.02 | 2.78 |
|  | S7 | Bottom | 8.1 | 27.88 | 24.00 | 38.33 | 0.10 | 6.79 | 1.47 | 5.94 | 3.01 |
|  | S8 | Surface | 21.5 | 27.56 | 30.28 | 12.85 | 0.13 | 6.07 | 1.69 | 6.75 | 0.68 |
|  | S8 | Bottom | 21.5 | 26.12 | 32.37 | 8.54 | 0.16 | 11.79 | 1.37 | 8.01 | 0.84 |
| Cruise | **Stations** | **Sample layer** | **Water depth (m)** | **Temperature(℃)** | **Salinity** | **DIN (μM)** | **PO_4_ (μM)** | **SiO_4_ (μM)** | **DOC (×10^3^ μg C /L)** | **DOC:DON ratio** | **Chl *a*(μg/L)** |
| January 2016 | S1 | Surface | 13.5 | 18.02 | 3.50 | 179.12 | 1.92 | 83.57 |  | 2.17 | 1.99 |
|  | S1 | Bottom | 13.5 | 18.13 | 4.63 | 181.76 | 1.90 | 83.95 | 1.87 | 1.92 | 1.76 |
|  | S2 | Surface | 18.5 | 18.32 | 8.06 | 170.43 | 1.99 | 80.85 | 1.53 | 2.14 | 1.19 |
|  | S2 | Bottom | 18.5 | 18.47 | 10.46 | 156.02 | 2.02 | 90.16 | 1.53 | 2.05 | 0.90 |
|  | S3 | Surface | 3.8 | 18.35 | 6.08 | 132.68 | 1.67 | 64.96 | 1.42 |  | 8.46 |
|  | S3 | Bottom | 3.8 | 18.47 | 9.40 | 118.08 | 1.41 | 75.04 |  | 3.65 | 4.54 |
|  | S4 | Surface | 13.5 | 19.26 | 20.05 | 105.03 | 1.61 | 62.64 | 1.25 | 3.50 | 0.86 |
|  | S4 | Bottom | 13.5 | 19.21 | 20.61 | 100.13 | 1.55 | 61.09 | 1.68 | 2.79 | 0.88 |
|  | S5 | Surface | 10.0 | 19.53 | 23.70 | 75.31 | 1.22 | 48.68 | 1.22 | 4.61 | 1.71 |
|  | S5 | Bottom | 10.0 | 19.53 | 24.32 | 69.51 | 1.26 | 52.22 | 1.62 | 3.42 | 1.83 |
|  | S6 | Surface | 7.0 | 18.89 | 23.50 | 66.59 | 1.08 | 43.26 | 1.15 | 4.78 | 3.21 |
|  | S6 | Bottom | 7.0 | 18.73 | 23.62 | 57.87 | 0.54 | 43.26 | 1.22 | 3.56 | 2.48 |
|  | S7 | Surface | 8.5 | 19.30 | 28.17 | 39.17 | 0.97 | 32.45 | 1.18 | 5.91 | 5.47 |
|  | S7 | Bottom | 8.5 | 19.20 | 28.45 | 31.37 | 0.92 | 27.80 | 0.92 | 10.05 | 4.58 |
|  | S8 | Surface | 21.5 | 19.69 | 32.61 | 8.54 | 0.28 | 12.64 | 1.11 | 10.68 | 1.57 |
|  | S8 | Bottom | 21.5 | 19.49 | 32.21 | 8.78 | 0.26 | 12.25 | 1.07 | 11.85 | 2.34 |

Table 2 BA, BP*_i_*, BP*_f_*, BP, sBP, BR, sBR, BCD and BGE at the surface and bottom at eight stations in the wet season (May 2015) and the dry season (January 2016).

| Cruises | | Stations | Sample layer | BA  (×10^9^ Cells/L) | BP*_i_*  (μg C L^-1^ d^-1^) | BP*_f_*  (μg C L^-1^ d^-1^) | BP  (μg C L^-1^ d^-1^) | sBP  (fg C Cell^-1^ d^-1^) | BR  (μg C L^-1^ d^-1^) | sBR  (fg C Cell^-1^ d^-1^) | BCD  (μg C L^-1^ d^-1^) | BGE  (%) |
| --- | --- | --- | --- | --- | --- | --- | --- | --- | --- | --- | --- | --- |
| May 2015 | | S1 | Surface |  | 17.110 | 226.951 | 119.79 |  | 223.11 | 194.13 | 342.90 | 34.93 |
|  | | S1 | Bottom |  | 16.476 | 277.296 | 144.07 |  | 236.43 | 156.12 | 380.50 | 37.86 |
|  | | S2 | Surface | 3.80 | 0.454 | 201.494 | 99.14 | 55.04 | 235.35 | 131.09 | 334.49 | 29.64 |
|  | | S2 | Bottom | 2.95 | 5.871 | 134.054 | 68.97 | 32.07 | 128.96 | 62.29 | 197.92 | 34.85 |
|  | | S3 | Surface | 4.19 | 0.531 | 226.828 | 111.44 | 43.33 | 181.94 | 72.90 | 293.38 | 37.98 |
|  | | S3 | Bottom | 3.29 | 3.736 | 159.235 | 79.88 | 43.90 | 93.63 | 51.46 | 173.50 | 46.04 |
|  | | S4 | Surface | 2.36 | 0.819 | 133.084 | 70.54 | 34.74 | 220.56 | 138.95 | 291.11 | 24.23 |
|  | | S4 | Bottom | 1.51 | 2.710 | 111.568 | 56.99 | 44.60 | 135.17 | 106.52 | 192.16 | 29.66 |
|  | | S5 | Surface | 1.85 | 1.098 | 220.677 | 110.27 | 50.33 | 152.10 | 69.30 | 262.37 | 42.03 |
|  | | S5 | Bottom | 2.33 | 1.090 | 83.536 | 51.84 | 30.97 | 66.59 | 48.35 | 118.43 | 43.77 |
|  | | S6 | Surface | 4.77 | 8.404 | 100.428 | 57.62 | 22.24 | 566.64 | 223.06 | 624.26 | 9.23 |
|  | S6 | | Bottom | 5.44 | 1.264 | 55.041 | 33.27 | 10.27 | 321.91 | 109.72 | 355.18 | 9.37 |
|  | S7 | | Surface | 2.23 | 0.131 | 60.705 | 33.75 | 34.12 | 148.49 | 149.85 | 182.24 | 18.52 |
|  | S7 | | Bottom | 3.50 | 0.137 | 81.765 | 38.50 | 15.92 | 172.74 | 72.51 | 211.24 | 18.23 |
|  | S8 | | Surface | 1.39 | 0.064 | 62.550 | 36.89 | 33.70 | 297.87 | 304.27 | 334.76 | 11.02 |
|  | S8 | | Bottom | 1.37 | 0.219 | 37.248 | 22.61 | 19.81 | 115.07 | 93.44 | 137.69 | 16.42 |
| Cruises | | **Stations** | **Sample layer** | **BA**  **(×10^9^ Cells/L)** | **BP*_i_***  **(μg C L^-1^ d^-1^)** | **BP*_f_***  **(μg C L^-1^ d^-1^)** | **BP**  **(μg C L^-1^ d^-1^)** | **sBP**  **(fg C Cell^-1^ d^-1^)** | **BR**  **(μg C L^-1^ d^-1^)** | **sBR**  **(fg C Cell^-1^ d^-1^)** | **BCD**  **(μg C L^-1^ d^-1^)** | **BGE**  **(%)** |
| January 2016 | | S1 | Surface | 1.75 | 0.592 | 16.872 | 16.73 | 37.92 | 103.91 | 235.34 | 120.64 | 13.87 |
|  | | S1 | Bottom | 1.81 | 0.608 | 13.644 | 13.59 | 27.00 | 114.57 | 227.26 | 128.16 | 10.60 |
|  | | S2 | Surface | 1.26 | 0.416 | 21.566 | 11.10 | 27.51 | 73.58 | 229.45 | 84.68 | 13.11 |
|  | | S2 | Bottom | 1.04 | 0.267 | 3.837 |  |  | 192.53 | 337.62 |  |  |
|  | | S3 | Surface | 1.16 | 0.366 | 45.356 | 22.86 | 49.25 | 110.06 | 236.75 | 132.92 | 17.20 |
|  | | S3 | Bottom | 6.66 | 0.124 | 16.316 | 16.57 | 29.18 | 104.26 | 383.78 | 120.83 | 13.71 |
|  | | S4 | Surface | 5.67 | 0.376 | 111.057 | 57.08 | 22.57 | 333.47 | 121.92 | 390.55 | 14.62 |
|  | | S4 | Bottom | 6.34 | 0.284 | 28.439 | 15.53 | 15.97 | 73.45 | 75.20 | 88.98 | 17.45 |
|  | | S5 | Surface | 1.23 | 0.326 | 4.610 | 18.08 | 20.71 | 235.57 | 269.47 | 253.65 | 7.13 |
|  | | S5 | Bottom | 1.11 | 0.534 | 1.453 | 11.01 | 17.86 | 99.47 | 158.88 | 110.48 | 9.97 |
|  | | S6 | Surface | 8.81 | 2.682 | 32.247 | 19.30 | 10.50 | 275.98 | 155.34 | 295.28 | 6.54 |
|  | | S6 | Bottom | 8.28 | 0.941 | 13.027 | 7.72 | 12.29 | 64.61 | 102.83 | 72.34 | 10.68 |
|  | | S7 | Surface | 9.10 | 0.377 | 27.098 | 15.83 | 9.34 | 337.32 | 230.20 | 353.14 | 4.48 |
|  | | S7 | Bottom | 1.01 | 0.362 | 8.160 | 4.92 | 6.08 | 86.42 | 91.69 | 91.34 | 5.39 |
|  | | S8 | Surface | 1.01 | 0.260 | 8.910 | 13.19 | 5.81 | 243.96 | 184.65 | 257.14 | 5.13 |
|  | | S8 | Bottom | 9.88 | 0.336 | 6.179 | 3.93 | 5.77 | 77.14 | 94.03 | 81.07 | 4.85 |

Table 3 The equations and correlation coefficients between various variables (DOC, DOC:DON ratio, Chl a, BA, BP, sBP, BR, BCD and BGE)

| Variables | Equations | Correlation coefficient (r^2^) |
| --- | --- | --- |
| DOC vs Chl *a* | Log (DOC) = 0.13 × Log (Chl a) + 3.13 | r^2^ = 0.19** |
| BA vs DOC | Log (BA) = 1.91 × Log (DOC) + 3.08 | r^2^ = 0.46** |
| BP vs DOC | Log (BP) = 4.79 × Log (DOC) – 13.84 | r^2^ = 0.20** |
| sBP vs DOC | Log (sBP) = 3.82 × Log (DOC) – 10.79 | r^2^ = 0.57** |
| BR vs DOC | Log (BR) = 3.77 × Log (DOC) – 9.97 | r^2^ = 0.26** |
| BCD vs DOC | Log (BCD) = 4.55 × Log (DOC) – 12.23 | r^2^ = 0.17* |
| BGE vs DOC | Log (BCD) = 3.80 × Log (DOC) – 10.96 | r^2^ = 0.47** |
| BP vs DOC:DON ratio | Log (BP) = -1.53 × Log (DOC:DON ratio) – 2.34 | r^2^ = 0.33** |
| sBP vs DOC:DON ratio | Log (sBP) = -1.08 × Log (DOC:DON ratio) + 1.96 | r^2^ = 0.61** |
| BGE vs DOC:DON ratio | Log (BP) = -1.16 × Log (DOC:DON ratio) + 1.84 | r^2^ = 0.54** |
| BA vs Chl *a* | Log (BA) = 0.41 × Log (Chl *a*) + 9.00 | r^2^ = 0.33** |
| BP vs Chl *a* | Log (BP) = 0.65 × Log (Chl *a*) + 1.15 | r^2^ = 0.17* |
| BR vs Chl *a* | Log (BR) = 0.40 × Log (Chl *a*) + 1.93 | r^2^ = 0.40** |
| BCD vs Chl *a* | Log (BCD) = 0.28 × Log (Chl *a*) + 2.15 | r^2^ = 0.17* |
| BP vs BA | Log (BP) = 2.26 × Log (BA) – 19.38 | r^2^ = 0.39** |
| BR vs BA | Log (BR) = 1.34 × Log (BA) – 10.30 | r^2^ = 0.40** |

Note: * and ** denote that variables were significant at 0.05 (*p* < 0.05) and 0.01 (*p* < 0.01) levels, respectively.
